# Prefrontal activation in bipolar and unipolar depression patients in the letter fluency tasks and category fluency tasks: a functional near-infrared spectroscopy study

**DOI:** 10.3389/fpsyt.2025.1610703

**Published:** 2025-09-25

**Authors:** Juan Hui, Haiyue Dai, Qi Lu, Juan Wang, Guimei Cui, Junlin Mu, Lin Zhao, Shina Gu, Juan Li, Zhaohui Zhang

**Affiliations:** ^1^ The Second Affiliated Hospital of Xinxiang Medical University (Henan Mental Hospital), Xinxiang, China; ^2^ Henan Key Laboratory of Neurorestoratology, The First Affiliated Hospital of Xinxiang Medical University, Weihui, Henan, China; ^3^ Henan Engineering Research Center of Physical Diagnostics and Treatment Technology for the Mental and Neurological Diseases, Xinxiang, China; ^4^ Shandong Mental Health Center, Jinan, China; ^5^ Henan Collaborative Innovation Center of Prevention and Treatment of Mental Disorders, Xinxiang, Henan, China

**Keywords:** letter fluency tasks, category fluency tasks, fNIRS, unipolar depression, bipolar depression

## Abstract

**Background:**

Distinguishing bipolar disorder (BD) from unipolar depression (UD) remains a critical clinical challenge. Improved diagnostic accuracy could enhance therapeutic outcomes for both conditions. This study aims to (1) identify functional near-infrared spectroscopy (fNIRS)-based biomarkers differentiating BD from UD, and (2) compare frontotemporal hemodynamic responses during phonological (LFT) and semantic (CFT) verbal fluency tasks.

**Methods:**

We recruited 100 participants: 33 with UD, 34 with BD, and 33 healthy controls (HC). Cortical oxygenation changes ([oxy-Hb]) were recorded using 52-channel fNIRS during LFT and CFT performance.

**Findings:**

The [oxy-Hb] activation in the UD and BD groups was lower compared to the HC group. Most channels demonstrated the [oxy-Hb] activation is lowest in BD patients, followed by UD patients, and the highest in the HC participants. Compared to CFT, UD and HC patients exhibited more extensive prefrontal cortex activation during LFT. This study found differences in [oxy-Hb] activation in the ventrolateral prefrontal cortex (VLPFC) between BD and UD patients during the CFT period.

**Conclusion:**

Our findings indicate that the LFT elicits more extensive prefrontal activation, with differential engagement of the VLPFC in BD compared to UD. These results suggest potential neuroimaging biomarkers for distinguishing between UD and BD, while also providing insights into the neural substrates of language processing.

## 1 Introduction

Bipolar disorder is a mental illness distinguished by the occurrence of mania (or hypomania) and severe depressive episodes alternating with periods of partial or complete remission. Its lifetime prevalence ranges from 2% to 5% (1, 2). Bipolar disorder is often persistent and recurrent, severely impairing an individual’s social function at school or work, which can affect the quality of life and increase suicide rates (3). Because first-episode types among bipolar disorder patients commonly manifest with depressive episodes, which are the most prevalent mood states in the disease course of bipolar disorder (4–6). Diagnosing unipolar depression (UD) and bipolar depression (BD) accurately in clinical practice is challenging. Prior studies indicate that 7%-52% of patients with BD are initially misdiagnosed with UD (7). Inaccurate diagnosis leads to inappropriate drug prescription and poor prognosis (8, 9). Although studies have found that patients with BD are characterized by a younger age of onset, more frequent depressive episodes, and more severe symptoms, and that patients with UD have a higher frequency of biological rhythm disturbances, it is difficult to differentiate between the two based on this characteristic (10). Therefore, searching for neuroimaging markers that correctly differentiate UD from BD is crucial for clinical guidance.

Cognitive impairment is an important psychopathological characteristic of UD (11) and BD (12). Many studies have demonstrated a wide range of cognitive deficits in both UD and BD, primarily in the areas of memory, attention, executive functioning and language (13, 14). UD and BD cognitive deficits persist during the onset, progression, and remission of the disease (15–18). UD and BD cognitive deficits differ in terms of both expression and severity. In contrast to UD, BD exhibits more severe cognitive deficits (19, 20). UD and BD have different linguistic profiles, with memory deficits appearing to be more significant in UD patients and executive dysfunction in BD patients (21). Studies consistently find that these cognitive deficits are linked to changes in the structure and function of prefrontal and temporal lobe regions (22–25).

Verbal fluency tasks (VFT) is one of the most commonly used neurocognitive measurement tools. VFT are commonly employed to assess cognitive impairment in psychiatric conditions, including depression, bipolar disorder, and schizophrenia, and are primarily used to assess verbal ability, memory, attention, and executive ability (26, 27). The VFT is utilized to assess the structural or functional integrity of the frontotemporal lobe because the neural substrates for these cognitive functions are primarily found in the prefrontal (PFC) or temporal cortex (28, 29). VFT consist of two categories: letter fluency tasks (LFT) and category fluency tasks (CFT). In these tasks, participants must produce as many words as possible using a given letter or within a specific semantic category within a time limit.

VFT is one of the most commonly activated tasks in functional near-infrared spectroscopy (fNIRS). fNIRS is a non-invasive imaging technique based on optical principles that reflects hemodynamic changes in the brain by measuring real-time changes in oxyhemoglobin (oxy-Hb) and deoxyhemoglobin (deoxy-Hb) (30). The fNIRS measurements of cortical oxy-Hb change parameters were in high concordance with blood oxygenation-level dependent (BOLD) from magnetic resonance imaging (MRI) (31). Compared with other neurofunctional imaging tools, fNIRS has the advantages of good temporal resolution, a wide range of applicability, insensitivity to motion, unlimited application scenarios, and a more comprehensive depiction of brain functions. Research has proven that fNIRS can assist doctors in diagnosing psychiatric diseases and distinguishing between different subtypes (32–35). Using fNIRS to study cerebral hemodynamic responses to BD and UD may provide insights into pathophysiology and diagnosis (36, 37).

Current research primarily focuses on cortical activation differences between healthy controls (HC) and patients with UD or BD (38–42), while direct comparisons between UD and BD remain scarce. A study has found that phonological and semantic tasks not only engage shared cognitive processes but may also involve distinct, task-specific mechanisms (43). A meta-analysis revealed specialized roles in phonological and semantic processing: the left inferior parietal lobule and right superior temporal gyrus for phonological processing, versus the left middle temporal gyrus for semantic processing. The left inferior frontal gyrus showed functional dissociation, with its posterior dorsal region supporting phonological processing and anterior ventral region involved in semantic processing (44). Gourovitch, M L et al. found the inferior frontal cortex and temporoparietal cortex showed greater activation during letter fluency compared to semantic fluency, whereas the left temporal cortex demonstrated the opposite pattern, with stronger activation during semantic fluency tasks (45). However, existing VFT studies on UD and BD have employed either CFT or LFT alone, but not both. Furthermore, the differential neural activation patterns in Chinese psychiatric patients during CFT and LFT remain unclear. This study aims to use fNIRS to investigate differences in brain activation characteristics among UD, BD, and HC groups during both CFT and LFT tasks. The main objective is to identify objective biomarkers for distinguishing BD and UD patients and to determine more sensitive tasks for assessing verbal fluency deficits in Chinese UD, BD, and HC populations.

## 2 Materials and methods

### 2.1 Participants

From July 2022 to September 2023, ninety-six patients were recruited from the outpatient and inpatient departments of the Second Affiliated Hospital of Xinxiang Medical College. This included 33 patients with UD and 34 with BD. Two senior physicians utilized the International Classification of Disease (ICD)-10 to diagnose all patients. All patients were assessed using the 17-item Hamilton Depression Rating Scale (HAMD-17), the 14-item Hamilton Anxiety Rating Scale (HAMA-14), the mood disorder questionnaire (MDQ), and the Young Mania Rating Scale (YMRS) before fNIRS. Both the UD and BD groups had HAMD-17 scores >17 and YMRS scores <6. The UD group had an MDQ score <5, while the BD group had an MDQ score >7. None of the patients had controlled medications, and most were taking medications throughout the duration of the study. Thirty-three healthy subjects were also selected from the local community and evaluated by an experienced psychiatrist to rule out psychiatric disorders. All subjects were right-handed, aged 18–60 years old, and had at least a middle school education.

Exclusion criteria for this study included: a history of traumatic brain injury or other organic brain diseases, major physical illnesses, or poor hearing; comorbidities, including other psychiatric disorders such as substance dependence and obsessive-compulsive disorder; pregnant and breastfeeding women; patients who had undergone non-convulsive electroconvulsive therapy (ECT) within the last six months; and patients who failed to finish the cognitive tasks or the fNIRS test.

The Ethics Committee of the Second Affiliated Hospital of Xinxiang Medical College (XYEFYLL-2022-42-2) approved this study, which was registered with the Chinese Clinical Trials Registry (ChiCTR2400088275). All subjects were thoroughly apprised of the study’s nature and process and voluntarily signed a written informed consent form.

### 2.2 Verbal fluency tasks

**Figure 1:**
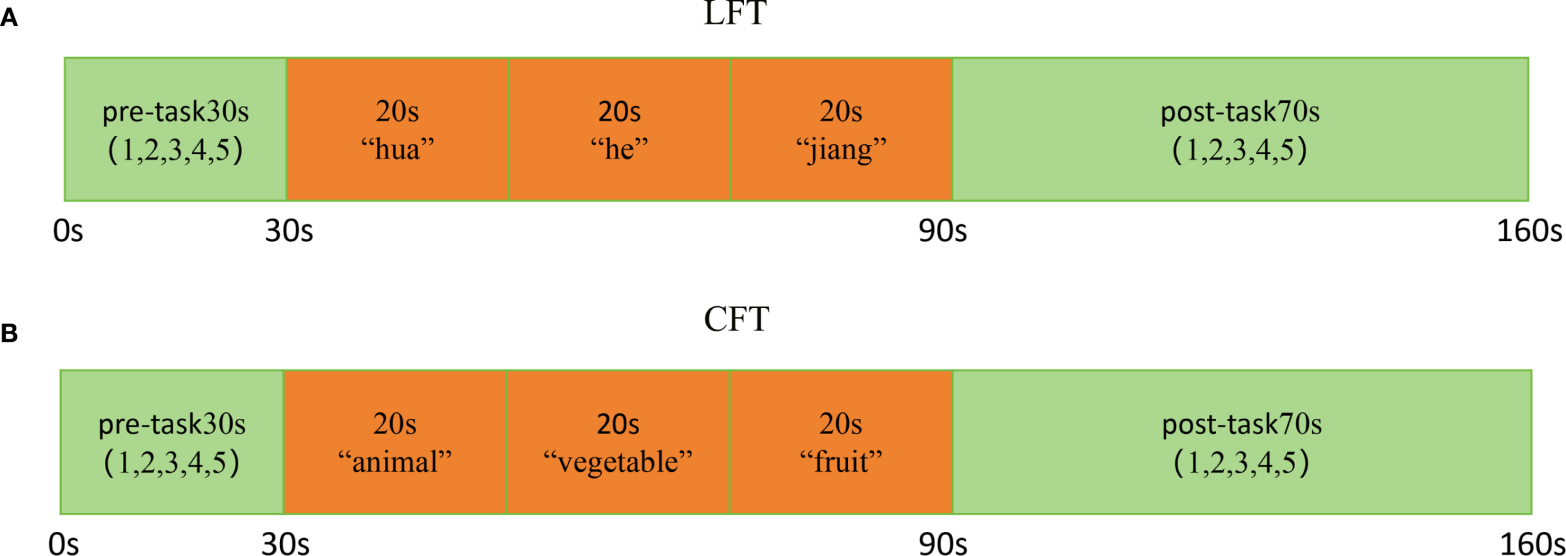
Activation task design. **(A)** LFT and **(B)** CFT. The activation task is divided into three phases: a 30s pre-task, a 60s task period, and a 70s post-task period.

The test was conducted by a well-trained and qualified person. Subjects maintained proper posture before the test and gazed at a red mark on the wall ahead to avoid excessive body or head movement. Prior to testing, subjects practiced with “大” to ensure they understood and could perform the task correctly. All subjects completed the LFT first, followed by the CFT. A 10-second delay was implemented before the test began to ensure the stability of the fNIRS waveform. Each test totaled 170s, consisting of a 30s pre-task period, a 60s task period, and a 70s post-task period (**Figure 1**). During the pre-task and task periods, subjects were instructed to repeat the numbers “1, 2, 3, 4, 5”. The LFT task required subjects to form as many words as possible using the words “花”, “河”, and “江”. During the CFT task, subjects were asked to say a maximum number of words belonging to the categories “animal”, “vegetable”, and “fruit”. The same researcher recorded the number of valid words produced by each subject. The quantity of valid words produced during the task indicates the subject’s performance on the task.

### 2.3 fNIRS measurement

**Figure 2:**
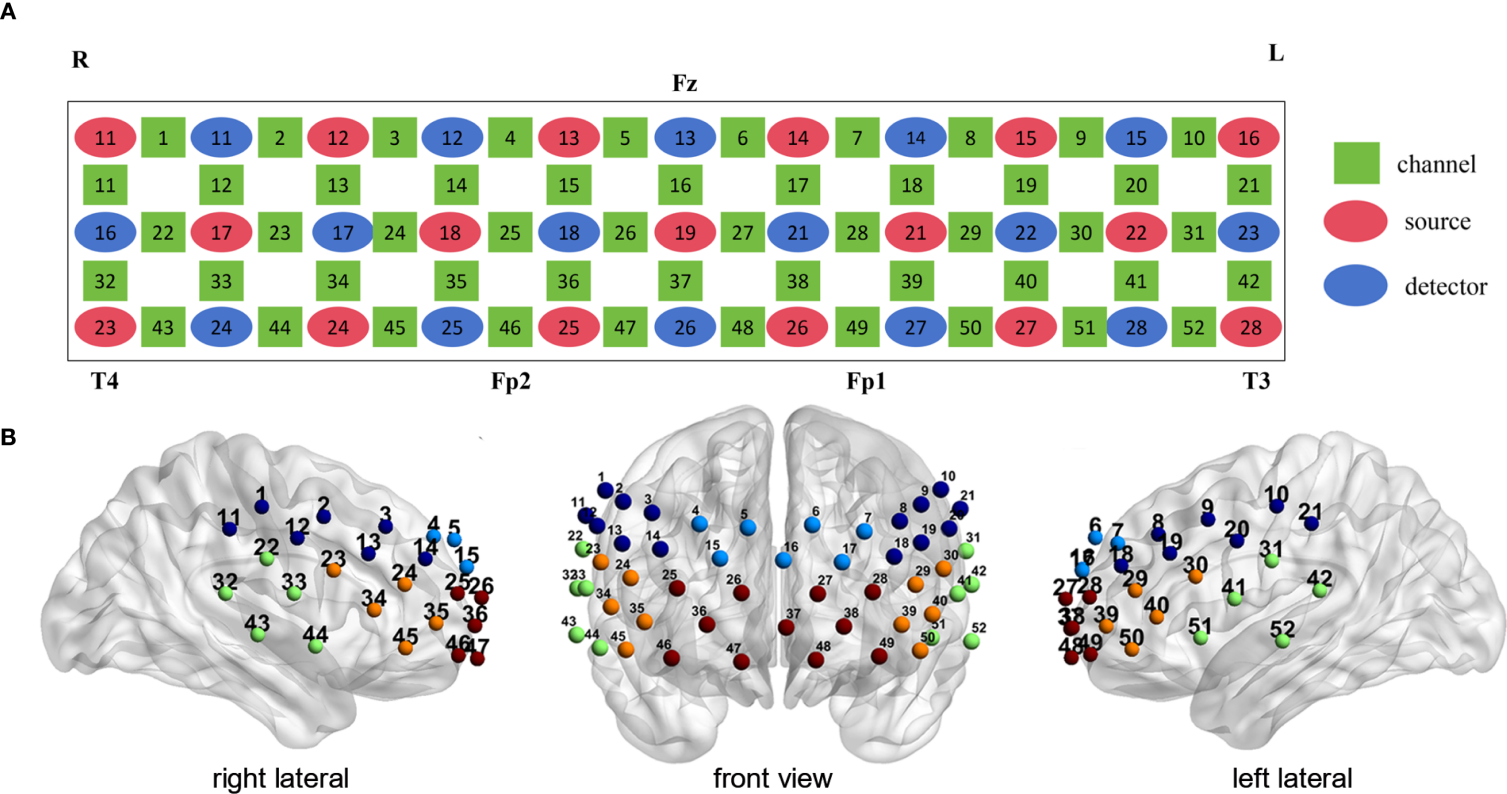
Probe positions and channel settings in 52-channel NIR near-infrared spectroscopy. **(A)** Channel arrangement according to the international 10–20 system. **(B)** Location of 8 Brodmann regions according to 52 channels (Light blue: dorsal frontal pole cortex. Red: ventral prefrontal cortex. Orange: ventrolateral prefrontal cortex. Dark blue: dorsolateral prefrontal cortex. Green: superior temporal gyrus).

Cortical activation during the LFT and CFT of subjects at two wavelengths (695 nm and 830 nm) was measured using a 52-channel fNIRS (ETG-4100, Hitachi Medical Corporation, Tokyo, Japan). The sampling frequency is 10 Hz. The 52-channel structure is distributed in a 3 x 11 arrangement and contains 17 transmitters and 16 detectors (**Figure 2A**). The transmitter and detector are 3 cm apart, and the area between them is defined as a channel. The fNIRS was placed in the frontal temporal lobe region of the subjects following the EEG 10–20 system (**Figure 2B**). Evaluating the spatial information of each channel in the Montreal Neurological Institute using NIRS_SPM (version 4.0) (46).

### 2.4 fNIRS data analysis

All NIRS data processing, from detrending to analysis, was performed using NIRS-SPM (https://www.nitrc.org/projects/nirs_spm/). Prior to further processing, the hemodynamic response function (HRF) and wavelet-minimum description length (MDL)-based detrending methods were applied to correct for global drift and eliminate systemic noise and physiological variations (47). NIRS channel timecourses were modeled in NIRS-SPM using a general linear model (GLM). The change in [oxy-Hb] and [deoxy-Hb] concentrations in each channel was calculated using a revised Beer-Lambert law. A channel-by-channel least squares estimation of the beta (β) value was performed to indicate the degree of regional activation during each subject’s task period. To analyze the near-infrared spectral data, the [oxy-Hb] change was utilized as activation data. Compared to changes in [deoxy-Hb], changes in [oxy-Hb] reflect cognitive activation more directly, as demonstrated by the higher correlation between BOLD changes in [oxy-Hb] measured by MRI (48). The false discovery rate (FDR) was utilized to correct for multiple comparisons (threshold set to *P*< 0.05) and to assess neuronal activation in channel 52 (49). Visualized brain networks were generated using BrainNet Viewer (50).

### 2.5 Statistical analysis

The three participant groups were analyzed for clinical variables using the Kruskal–Wallis test. A rank-based analysis of covariance (ANCOVA) was conducted to compare behavioral performance and fNIRS data between the three groups during CFT and LFT tasks, adjusting for gender and age as covariates. We applied the Bonferroni correction to correct multiple comparisons and performed modified p-values for any *post-hoc* tests to compare between any two specific groups. We employed the non-parametric one-sample Wilcoxon signed-rank test to compare the original signals with a median of 0 (null hypothesis value), assessing the activation significance for individual fNIRS channels and brain regions. Use the chi-square test to compare the differences in the number of activated channels between CFT and LFT. Differences in categorical variables among the three groups of participants were assessed using chi-square tests. The Wilcoxon 2-related samples test was used when comparing differences in fNIRS activation in the same brain regions under CFT and LFT in the same groups of people. And corrected for multiple comparisons using the false discovery rate (FDR), *P*< 0.05 indicating a statistical difference. All statistical analyses in this study were conducted using SPSS 25.0.

## 3 Result

### 3.1 Demographic characteristics

**Table 1** presents the demographic and clinical characteristics of all participants. No significant differences were observed among the three groups of subjects concerning age and years of education and family history. There was no significant difference in onset age of illness, duration of illness, HAMD scores and HAMA scores between BD and UD (HAMD: *Z* = 3.314, *P* = 0.632; HAMA: *Z* = 4.658, *P* = 0.510). Compared with the UD group, the BD group was more male.

**Table 1:** Demographic and clinical characteristics of the three groups.

	UD (n=33)	BD (n=34)	HC (n=33)	*X^2^/Z/H/F*	*P*
Gender (male/female)	6/27	26/8	9/24	27.358	<0.001
Age (years)	27(21,37.5)	24.5(20.5,35)	39 (24,45)	5.688	0.058
Years of education (years)	13(10.5,15)	12(11.75,15)	12 (12,15)	0.975	0.614
Onset age of illness (years)	23(16.5,29.5)	20(16,25.25)		1.278	0.258
Duration of illness (years)	5 (3,9)	5(2,9.25)		0.189	0.664
CFT	22(18,24.5)	20.5 (14,25)	26(23.5,28)	10.350	<0.001
LFT	12(6.5,15)	7.5 (4,10)	13(9.5,14)	6.140	0.003
HAMD	19 (18,22)	18 (18,22)	0 (0,1)	69.015	<0.001
HAMA	18(15.5,22)	17 (15,19)	0(0,1.5)	66.549	<0.001

BD, bipolar depression group; UD, unipolar depression group; HC, healthy controls group;CFT, Category Fluency Task; LFT, Letter Fluency Task; HAMD, Hamilton Depression Rating Scale, 17-item version, HAMA, Hamilton Anxiety Rating Scale.

### 3.2 VFT performance

When gender and age were included as covariates, the three groups showed significant differences in the number of words produced in the CFT (**Table 1**). Both the UD group and the BD group generated significantly fewer words compared to the HC group. The number of words produced by the UD and BD groups during the CFT showed no statistically significant difference. After controlling for gender and age, the BD group showed significantly lower LFT scores compared to the HC group (*P* = 0.012). Compared with the UD group, neither the BD nor the HC group showed statistically significant differences in LFT word generation performance. The CFT performance was better than the LFT performance in all three groups of subjects (HC: *Z*=-5.017, *P*<0.01; BD: *Z*=-5.012, *P*<0.01; UD: *Z*=-4.491, *P*<0.01).

### 3.3 Hemodynamic response during VFT

**Figure 3:**
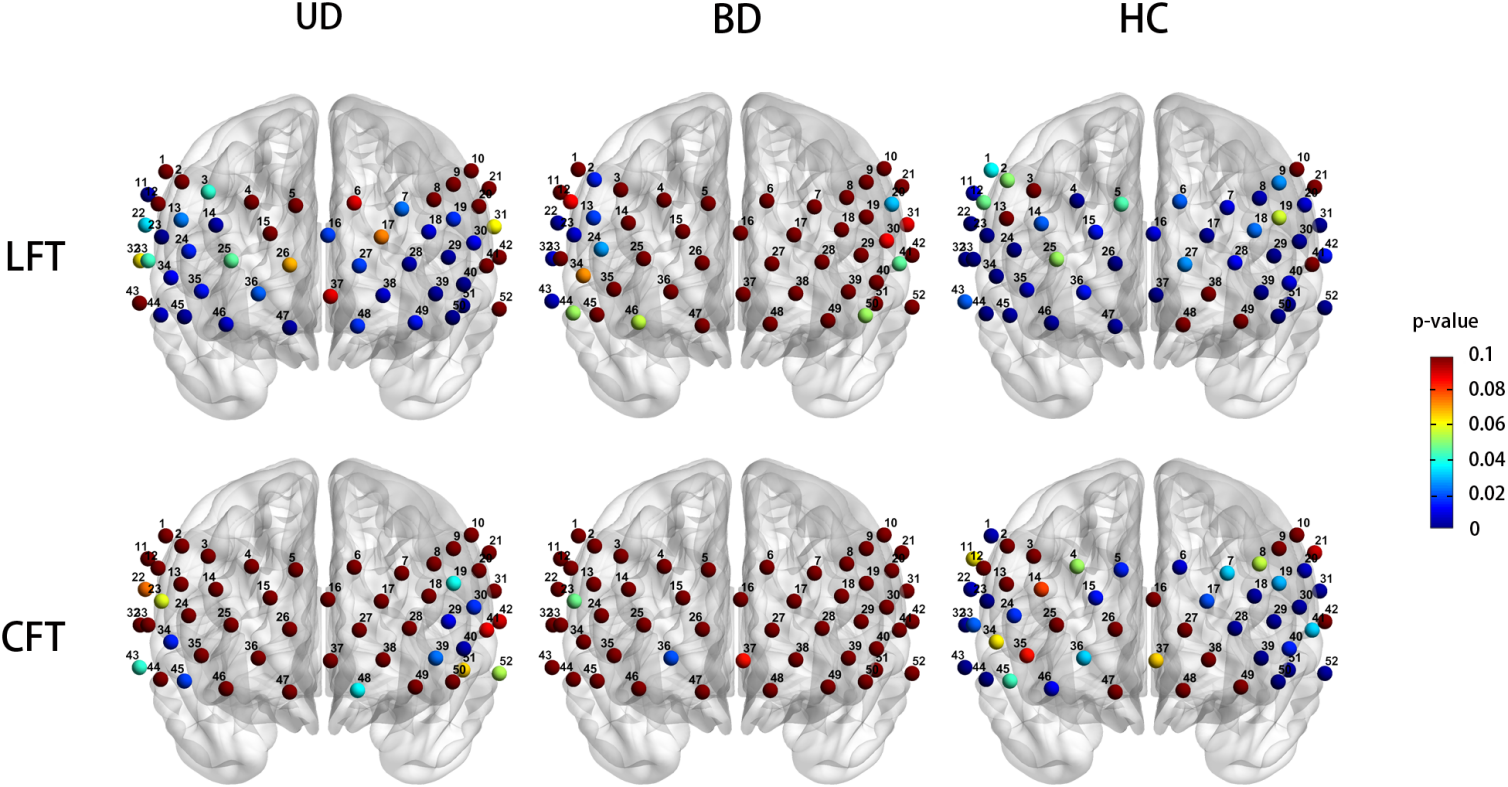
Cortical activation patters in the unipolar depression group (left column), bipolar depression group (middle column), and healthy controls group (right column) during the LFT (top row) and CFT (bottom row). Statistical significance (p < 0.05) is indicated by the color gradient shown in the right-side legend bar. LFT, letter fluency tasks; CFT, category fluency tasks; BD, bipolar depression group; UD, unipolar depression group; HC, healthy controls group.

During the CFT, the HC group exhibited significant activation in 23 channels (ch1, 5, 6,15, 17, 18, 20, 22-24, 28-33, 39, 40, 43, 44, 46, 50-52, FDR P < 0.05, 18 channels over the left hemisphere and 19 channels over the right hemisphere). No significant channel activation was observed in either the UD or BD group during the CFT (**Figure 3**).

During the LFT, significant activation was observed in 27 channels in the UD group (ch7, 11, 13, 14, 16, 18, 19, 23, 24, 27-30, 34-36, 38-40, 44-51, FDR *P* < 0.05, 14 channels over the left hemisphere and 12 channels over the right hemisphere, excluding medial channel 16), whereas no activation was detected in the BD group. The HC group exhibited significant activation in 39 channels during the CFT (ch1, 4, 6-9,11, 14-18, 20, 22-24, 26-37, 39, 40, 42-47, 50-52, FDR *P* < 0.05, 18 channels over the left hemisphere and 19 channels over the right hemisphere, excluding medial channel 37,16) (**Figure 3**).

### 3.4 Comparison of NIRS activation

**Figure 4:**
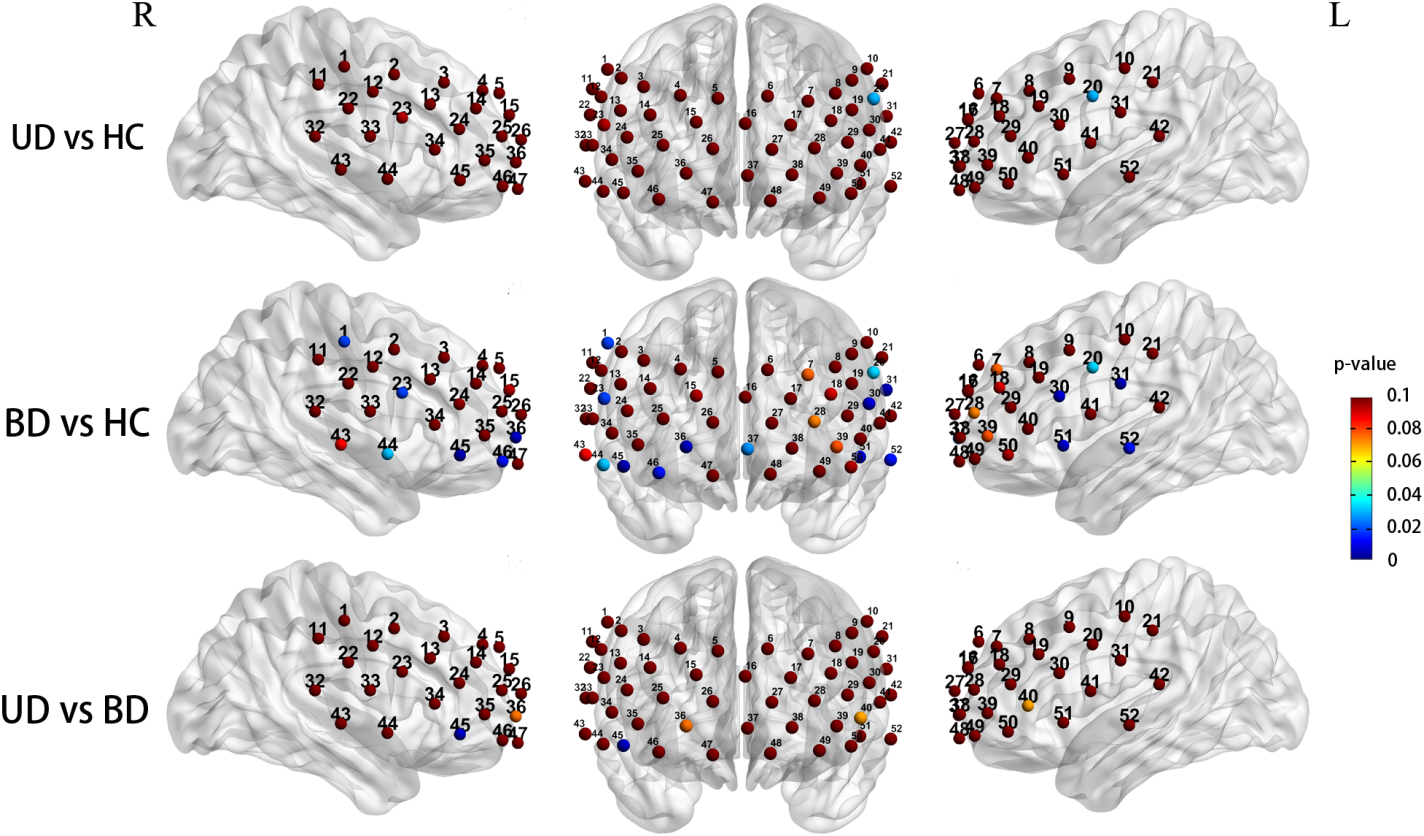
Group differences in task-related cortical activation during the CFT (Statistical significance (p < 0.05) is indicated by the color gradient shown in the right-side legend bar); R, right; L, left; BD, bipolar depression group; UD, unipolar depression group; HC, healthy controls group.

After controlling for gender and age as covariates, the three groups exhibited significantly different prefrontal activation patterns in the 12 channels during the CFT task (ch1, 20, 23,30, 31, 36, 37, 44, 45, 46, 51, 52; *F* = 3.633-7.087, *P* = 0.001-0.030). These channels correspond to the dorsolateral prefrontal cortex (DLPFC, ch1, 20, *F* = 4.513,4.823, *P* = 0.013,0.010), ventral prefrontal cortex (VFPC, ch36, 37, 46, *F* = 3.633-5.308, *P* = 0.007-0.030), superior temporal cortex (STC, ch31, 44, 51, 52, *F* = 3.935-5.806, *P* = 0.004-0.023), and ventrolateral prefrontal cortex (VLPFC, ch23, 30, 45, *F* = 4.587-7.087, *P* = 0.001-0.013). Compared to the HC group, BD subjects exhibited significantly reduced Oxy-Hb activation in the DLPFC (ch1, 20, *P* = 0.018, 0.032), VFPC (ch36, 37, 46, *P* = 0.005-0.026), STC (ch31,44,51,52, *P* = 0.003-0.030), and VLPFC (ch23,30,45, *P* = 0.003-0.017). The UD group exhibited significantly lower Oxy-Hb activation levels in the left DLPFC region compared to the HC group (ch20, *P* = 0.029). Compared with the UD group, BD patients showed notably reduced Oxy-Hb alterations in the right VLPFC (ch45, *P* = 0.004) (**Figure 4**).

**Figure 5:**
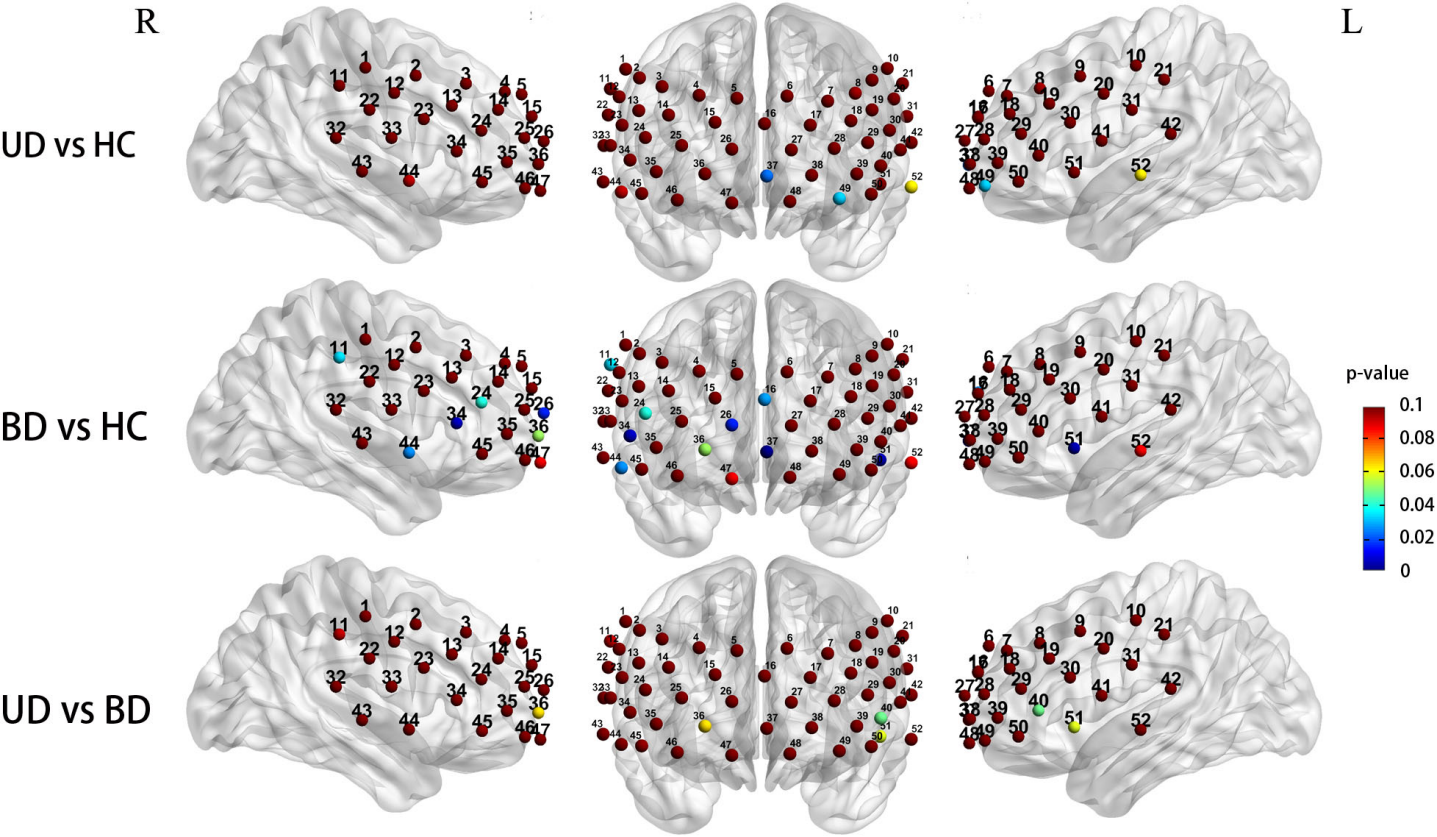
Group differences in task-related cortical activation during the LFT (Statistical significance (p < 0.05) is indicated by the color gradient shown in the right-side legend bar); R, right; L, left; BD, bipolar depression group; UD, unipolar depression group; HC, healthy controls group.

When controlling for gender and age covariates during the LFT, the three groups demonstrated significantly different prefrontal activation patterns in 11 functional channels (ch11, 16, 24, 26, 34, 36, 37, 44, 49, 51, 52; *F* = 3.194-8.893, *P* = 0.001-0.045). These channels respond to the DLPFC (ch11, *F* = 3.691, *P* = 0.029), DFPC (ch16, *F* = 3.585, *P* = 0.032), VFPC (ch26, 36, 37,49, *F* = 3.442-8.893, *P* = 0.001-0.036), VLPFC (ch24,34, *F* = 3.194,5.281, *P* = 0.045,0.007), STC (ch44,51,52, *F* = 3.597-5.883, *P* = 0.004-0.031). Compared to the HC group, UD participants showed significantly reduced Oxy-Hb changes in the VFPC (ch37,49, *P* = 0.022, 0.031),while BD patients exhibited significantly lower Oxy-Hb activation in the DFPC (ch16, *P* = 0.027), VFPC (ch26, 37, *P* = 0.014,0.001), bilateral STC (ch44, 51, *P* = 0.025, 0.003), the right DLPFC(ch11, *P* = 0.034), and the right VLPFC (ch24, 34, *P* = 0.039, 0.005). Comparisons between UD and BD groups revealed no significant differences in Oxy-Hb activation levels within prefrontal regions (**Figure 5**).

### 3.5 Comparison of VFTs activation

A comparison of activated channels in two VFT tasks showed that the UD and HC groups had more extensive activation during the LFT. Comparing fNIRS activation in the same brain regions under CFT and LFT within the same groups of people, we found that 4 channels (ch9, 11, 20, 33, P = 0.018-0.045) had higher activation values during LFT than during CFT in the UD group. Similarly, in the BD group, the activation values of 5 channels (ch2, 31, 36, 46, and 47, P = 0.019- 0.027) were higher during LFT than during CFT. The HC group showed higher activation values for channels ch26 and ch34 during LFT compared to CFT (P = 0.030, 0.021). However, these differences were not significant after FDR correction (FDR *P* < 0.05).

## 4 Discussion

To our knowledge, this is the first fNIRS study to compare cortical activation between UD and BD patients during CFT and LFT. The main findings can be summarized as follows (1): Both BD and UD patients exhibited frontal-temporal lobe impairments during both CFT and LFT (2). LFT induced broader prefrontal activation compared to CFT (3). BD patients demonstrated lower activation in the frontal and temporal regions compared to HC and UD groups (4). BD patients had lower activation in the VLPFC than UD patients.

This study found that patients with BD and UD had fewer words produced in CFT compared to HC group. There was no significant difference between BD and UD. The finding aligns with several earlier NIRS studies (42, 51, 52). Feng et al. found that no notable disparities were identified in CFT performance between UD and BD patients (33). The results demonstrated significantly impaired LFT performance in BD patients compared to healthy controls (HC), whereas neither BD nor HC groups showed statistically significant differences relative to the UD group. Some studies have found that UD patients have a fewer number of words in LFT than HC group (40, 53–55), Noda et al. found that there was no significant difference in LFT scores between the HC and UD groups (56). Inconsistent results may be partly attributed to the heterogeneity of depressed individuals and differences in task settings. The VFT results illustrate variations among the three groups in terms of working memory, executive functioning, language ability. According to VFT performance, symptoms of depression generally undermine executive functioning in the BD and UD groups. In contrast to LFT, all three groups produced a greater number of words during CFT. Charchat-Fichman et al. considers the executive function requirements of LFT to be higher than CFT. The internal connectivity of the semantic network during the CFT can help subjects search for words based on topic terms. During the LFT, subjects can only search for words independently without categorical cues (57). Schmidt et al. believes that knowledge is organized in the form of semantic networks, so CFT is usually easier than LFT (43). This result possibly suggests that the CFT advantage observed in the HC group was also preserved in the patient groups, consistent with previous findings.

Our study found that both the UD group and the HC group had more activated channels in the LFT compared to the CFT. Dan et al. found that LFT leads to broader activation (58). A meta-analytic review found that CFTs are much greater than LFT deficits in UD patients (59). Baldo et al. found that activation was lower during CFT than during LFT in patients with UD (60). The more severe semantic defects compared to phonetic defects in UD may be the reason why LFT activation is more extensive than CFT. Another possible reason is that Chinese characters, as logograms, contain semantic information within their phonetic components (61, 62). In LFT tasks, subjects might first search within a given category before shifting to another. Since LFT engages both phonological and semantic brain regions, it results in broader activation compared to CFT. Certainly, this rationale may not be applicable to alphabet-based languages such as English, as English verbal fluency tasks (VFT) may lack such intrinsic semantic or phonological associations. Moreover, CFT is generally simpler than LFT, as LFT requires the activation of more brain regions associated with cognitive functions (43).

Compared to HC, UD and BD patients exhibited decreased frontotemporal activation during CFT and LFT. This finding is consistent with previous studies (39, 63, 64), suggesting that both UD and BD are associated with frontotemporal impairments. Compared to HC, patients with UD exhibited reduced activation in the left DLPFC during the CFT and hypoactivation in the VFPC during the LFT. During CFT, BD patients showed hypoactivation across 12 channels (vs. HC), predominantly in the bilateral STG, VLPFC, DLPFC, and VFPC. During LFT, 8 channels exhibited reduced activation in BD, including bilateral STG, right DLPFC and VLPFC, VFPC, and DFPC. These brain regions are closely associated with human neuropsychological functions: DLPFC is linked to working memory and executive control (65), VLPFC plays a key role in emotional regulation (66), VFPC is involved in affective decision-making (67), STG is critical for language processing (68). In the present study, we found that among the channels with differences, [oxy-Hb] activation values were higher in the HC group compared to the UD group, and these values were higher than those in the BD group. A plausible explanation is that cognitive deficits exist in UD and BD patients (39, 63, 64), and cognitive deficits were more severe in BD compared to UD. This is compatible with previous findings (19, 20). Compared to UD patients, BD patients appear to exhibit more extensive and severe cognitive impairments, whereas UD patients seem to demonstrate significant deficits primarily in processing speed (69). Niu et al. discovered decreased cortical thickness in the dorsolateral prefrontal, frontal pole, and superior frontal gyrus in patients with BD compared to UD (70). Hermens et al. were also found that verbal memory and executive function were more severely impaired in BD patients (71).

In this study, we found differences in [oxy-Hb] activation in VLPFC between BD and UD patients during CFT. And no statistically significant difference was observed between UD and BD during the LFT. This may be attributed to the fact that CFT relies more heavily on temporal lobe functions (72), and cognitive deficits related to verbal fluency in bipolar disorder are associated with alterations in temporal network connectivity (37). In contrast, LFT depends more on regions such as the frontal lobe and is therefore less affected. Previous studies have also indicated that overall verbal fluency and executive function are more impaired in bipolar depression than in UD, though this difference is primarily reflected in CFT performance (73). Additionally, patients with BD exhibit more severe impairment in CFT than in LFT (74). Previous studies have found significant differences in VLPFC in UD and BD patients (5, 33, 75). The VLPFC has been recognized as an area dedicated to the processing and integration of social communication information (76), and the VLPFC is associated with emotional information integration and emotional response regulation (66). VLPFC is crucial in down-regulating emotional responses to social exclusion (77), and VLPFC abnormalities may affect interpersonal relationships and social adjustment (78). As a result, people with BD may have malfunctions in emotional regulation and integration, as well as interpersonal and social adjucstment. A review of neuroimaging studies on bipolar disorder found that altered functioning within and functional coupling between the ventrolateral prefrontal cortex and amygdala may represent a neural mechanism for the emotion dysregulation that characterizes bipolar disorder, given the key roles of these regions in emotion regulation. The researchers observed abnormally decreased activity in the ventrolateral prefrontal cortex (VLPFC) and orbitofrontal cortex (OFC) during emotion regulation (79). For the identification of UD and BD, there is potential for further research in the area.

## 5 Conclusion

This study compared the differences in [oxy-Hb] activation across three distinct groups of subjects during CFT and LFT. LFT induced more widespread activation than CFT, while BD patients showed consistently reduced activation in both tasks. The area with the greatest difference between the BD and UD groups is the VLPFC. We believe that the combination of fNIRS and a VFT task has the potential to be a valuable tool for enhancing the diagnostic precision in differentiating between UD and BD. The VLPFC has the potential for further research as a brain region that distinguishes between BD and UD. Future studies with larger and more diverse populations will be essential to validate these findings and elucidate the distinct neurobiological mechanisms underlying: VLPFC differentiation between UD and BD patients, and language-related impairments in these disorders.

## 6 Limitations

The current study has many limitations. First, the sample size of this study was insufficient in that it did not allow for a comparison of the hemodynamic responses of patients with different subtypes of the depressive phase of bipolar disorder. Second, the patient groups were all taking medications during the study period, and medications may affect fNIRS results. Previous studies have demonstrated that there was no relationship between antidepressant dose and Oxy-Hb activation in the BD and UD groups. The absence of a washout period or medication-naïve subgroup remains a limitation in this study. Third, there was a difference in gender distribution between the UD and BD groups. All participants in this study were in relatively stable condition, which may differ from patients in the acute phase of an attack, and this requires further research. Finally, the fixed order (LFT followed by CFT) in this study could potentially bias the activation patterns.

## Data Availability

The datasets presented in this study can be found in online repositories. The names of the repository/repositories and accession number(s) can be found below: the Chinese Clinical Trials Registry (ChiCTR2400088275).
