# Supplementary material for: Prefrontal activation in bipolar and unipolar depression patients in the letter fluency tasks and category fluency tasks: a functional near-infrared spectroscopy study
Source: Front Psychiatry. 2025 Sep 25;16:1610703. doi: 10.3389/fpsyt.2025.1610703 (PMC12507886; doi:10.3389/fpsyt.2025.1610703)
Supplement: Supplementary file 1 [file Table1.docx]

Supplementary Material

Correspondence between channels and brain regions. The MNI coordinates and anatomical locations corresponding to each channel.

| Channel | X | Y | Z | Anatomical label |
| --- | --- | --- | --- | --- |
| 1 | 62 | -15.67 | 49.33 | Right dorsolateral prefrontal cortex |
| 2 | 55.67 | 9.33 | 45.33 | Right dorsolateral prefrontal cortex |
| 3 | 45.33 | 34.33 | 41.33 | Right dorsolateral prefrontal cortex |
| 4 | 69 | -28.67 | 40.33 | dorsal frontal pole cortex |
| 5 | 65 | -1.33 | 36.67 | dorsal frontal pole cortex |
| 6 | 56 | 27.33 | 30.33 | dorsal frontal pole cortex |
| 7 | 42.33 | 50.33 | 28.33 | dorsal frontal pole cortex |
| 8 | 28.33 | 53.67 | 37.33 | Left dorsolateral prefrontal cortex |
| 9 | 11 | 62 | 36 | Left dorsolateral prefrontal cortex |
| 10 | -12 | 60.67 | 37 | Left dorsolateral prefrontal cortex |
| 11 | -30.67 | 51.67 | 34.67 | Right dorsolateral prefrontal cortex |
| 12 | 21 | 67 | 25 | Right dorsolateral prefrontal cortex |
| 13 | -1.67 | 66 | 24.33 | Right dorsolateral prefrontal cortex |
| 14 | -22.67 | 66 | 23.67 | Right dorsolateral prefrontal cortex |
| 15 | -43.33 | 35.33 | 38.33 | dorsal frontal pole cortex |
| 16 | -51 | 15.33 | 44.33 | dorsal frontal pole cortex |
| 17 | -58 | -12.33 | 49.67 | dorsal frontal pole cortex |
| 18 | -41.33 | 50.33 | 25.67 | Left dorsolateral prefrontal cortex |
| 19 | -51 | 30.67 | 30.67 | Left dorsolateral prefrontal cortex |
| 20 | -61 | 3.67 | 35.67 | Left dorsolateral prefrontal cortex |
| 21 | -65 | -26.33 | 42.67 | Left dorsolateral prefrontal cortex |
| 22 | 70 | -13.67 | 28.33 | Right superior temporal gyrus |
| 23 | 72 | -30.33 | 14.33 | Right dorsolateral prefrontal cortex |
| 24 | 69 | -2.67 | 14.33 | Right dorsolateral prefrontal cortex |
| 25 | 72.33 | -17.33 | -2.33 | ventral prefrontal cortex |
| 26 | 64 | 6 | -7 | ventral prefrontal cortex |
| 27 | 63.67 | 13.33 | 23.67 | ventral prefrontal cortex |
| 28 | 53 | 42 | 18 | ventral prefrontal cortex |
| 29 | 60 | 29.67 | 7.67 | Left dorsolateral prefrontal cortex |
| 30 | 48 | 54.67 | 2.33 | Left dorsolateral prefrontal cortex |
| 31 | 54.67 | 42.33 | -7.67 | Left superior temporal gyrus |
| 32 | 36.33 | 63.33 | 14.33 | Right superior temporal gyrus |
| 33 | 13.33 | 73 | 12.67 | Right superior temporal gyrus |
| 34 | -14 | 72.67 | 12.33 | Right dorsolateral prefrontal cortex |
| 35 | -33.67 | 63 | 13 | Right dorsolateral prefrontal cortex |
| 36 | 25.67 | 70.33 | 1.33 | ventral prefrontal cortex |
| 37 | -2.67 | 70.67 | 0.33 | ventral prefrontal cortex |
| 38 | -23.33 | 70 | 0.67 | ventral prefrontal cortex |
| 39 | 38.33 | 63.67 | -10.67 | Left dorsolateral prefrontal cortex |
| 40 | 13.33 | 71.33 | -12 | Left dorsolateral prefrontal cortex |
| 41 | -13.33 | 70.33 | -11.33 | Left superior temporal gyrus |
| 42 | -36 | 62.67 | -10 | Left superior temporal gyrus |
| 43 | -49 | 44.67 | 15.67 | Right superior temporal gyrus |
| 44 | -59 | 20.33 | 21.33 | Right superior temporal gyrus |
| 45 | -44 | 56.33 | 1.33 | Right dorsolateral prefrontal cortex |
| 46 | -55 | 36 | 5 | ventral prefrontal cortex |
| 47 | -50.67 | 46 | -8 | ventral prefrontal cortex |
| 48 | -67 | -10.33 | 27.67 | ventral prefrontal cortex |
| 49 | -64 | 4.67 | 12.33 | ventral prefrontal cortex |
| 50 | -69 | -29.67 | 15.67 | Left dorsolateral prefrontal cortex |
| 51 | -54.67 | 18.33 | -3.33 | Left superior temporal gyrus |
| 52 | -69 | -14.67 | -4.67 | Left superior temporal gyrus |
